# Sulcus Placement of PreserFlo Microshunt in Iridocorneal Endothelial Syndrome: A Case Report

**DOI:** 10.1155/crop/7474280

**Published:** 2026-09-30

**Authors:** Khizar Rana, Cindy Shi, Stephanie Stavrinakis, Soo Khai Ng

**Affiliations:** ^1^ Discipline of Ophthalmology and Visual Sciences University of Adelaide Adelaide South Australia Australia adelaide.edu.au; ^2^ Department of Ophthalmology Queen Elizabeth Hospital Adelaide South Australia Australia qehconnect.com; ^3^ Vision Eye Institute Kurralta Park Adelaide South Australia Australia

**Keywords:** angle closure glaucoma, ICE syndrome, PreserFlo Microshunt

## Abstract

**Background:**

Secondary angle‐closure glaucoma associated with iridocorneal endothelial (ICE) syndrome can be challenging to manage. This report presents a novel case of sulcus placement of a PreserFlo Microshunt in a patient with ICE syndrome following the failure of previous trabeculectomy and medical therapy.

**Case Presentation:**

A 61‐year‐old patient with secondary angle‐closure glaucoma due to ICE syndrome had uncontrolled intraocular pressures despite maximal medical therapy. They had a previous trabeculectomy, which failed following cataract surgery. Because of extensive peripheral anterior synechiae and risk of corneal decompensation, a PreserFlo Microshunt was placed in the sulcus. At the 18‐month postoperative follow‐up, intraocular pressure was well controlled without topical therapy.

**Conclusion:**

This is the first reported case of a PreserFlo Microshunt implanted in the sulcus in ICE syndrome. Sulcus placement provides a viable alternative in pseudophakic patients with extensive peripheral anterior synechiae, helping to mitigate the risk of corneal decompensation while effectively controlling intraocular pressure.

## 1. Introduction

Iridocorneal endothelial (ICE) syndrome is a group of conditions characterised by the migration of membranes of endothelial cells across the anterior chamber angle and onto the iris surface. These mechanisms can lead to tissue contracture, angle obstruction and the development of secondary angle closure glaucoma. Angle closure glaucoma with raised intraocular pressure (IOP) refractory to medical therapy has commonly been managed with trabeculectomy. However, ICE‐induced glaucoma is associated with higher failure rates of trabeculectomy than most other types of glaucoma [1]. PreserFlo Microshunt (Santen, Osaka, Japan) is an 8.5 mm long stent with an internal lumen to 70 *μ*m that can reduce IOP by creating a drainage pathway for the aqueous humour from the anterior chamber to the subconjunctival space. One previous case report describes the successful use of the PreserFlo Microshunt in ICE syndrome, and another describes PreserFlo in the sulcus in pseudoexfoliation glaucoma [2, 3]. Extensive peripheral anterior synechiae (PAS) and the risk of corneal decompensation in ICE syndrome may necessitate a modified approach to conventional placement in the anterior chamber. We herein describe the first successful placement of the PreserFlo Microshunt in the sulcus in a 61‐year‐old patient with secondary angle closure glaucoma.

## 2. Case Presentation

A 61‐year‐old female with a history of angle closure glaucoma secondary to essential iris atrophy presented with progressive vision loss in her right eye due to cataract. She had undergone a trabeculectomy 28 years earlier and was using Travoprost/Timolol once daily. On examination, her visual acuity in the right eye was 6/120 and 6/6 in the left eye. Goldman applanation IOPs were 14 mmHg and 12 mmHg in the right and left eyes, respectively, with central cornea thicknesses of 476 *μ*m and 524 *μ*m. She had a right superonasal shallow bleb, right cataract (NO5NC5P2 using Lens Opacities Classification System III) and extensive high PAS on gonioscopy. Her cup to disc ratio was 0.8 in the right eye and 0.4 in the left eye. Optical coherence tomography and Humphrey visual fields are shown in Figure 1. The patient underwent right cataract extraction and intraocular lens implantation, along with removal of iris obscuring the visual axis (Figure 2). Postoperatively, her visual acuity improved in the right eye to 6/15; however, the IOP increased to 32 mmHg on Travoprost/timolol. Brinzolamide and Brimonidine were added to the regimen; however, the IOP remained elevated at 26 mmHg. The trabeculectomy bleb was flat and scarred down. The patient consented to a redo filtration surgery with a PreserFlo Microshunt and mitomycin C 0.04%. Under sterile conditions, Tenon′s and conjunctiva were dissected from the sclera. Mitomycin 0.04% was applied inside the conjunctival/Tenon′s pocket for a total of 180 s. The sponges were then removed, and the conjunctiva and Tenon washed with balanced salt solutions. A 2.5‐mm mark from the anatomical limbus was made. A scleral tunnel was made with a 1‐mm angle knife, followed by 25‐Gauge needle with the needle aimed horizontally just behind the iris plane. The sulcus location was confirmed by direct visualisation of the needle tip entry behind the iris. The PreserFlo Microshunt was inserted bevel up, and position checked with gonioscopy (Figure 3). Tenon’s capsule and conjunctiva were closed with 10‐0 Nylon sutures. Subconjunctival dexamethasone was injected at the end of the case. Postoperatively, all hypotensive drops were stopped, and the patient commenced on a tapering regime of Maxidex.

**Figure 1:**
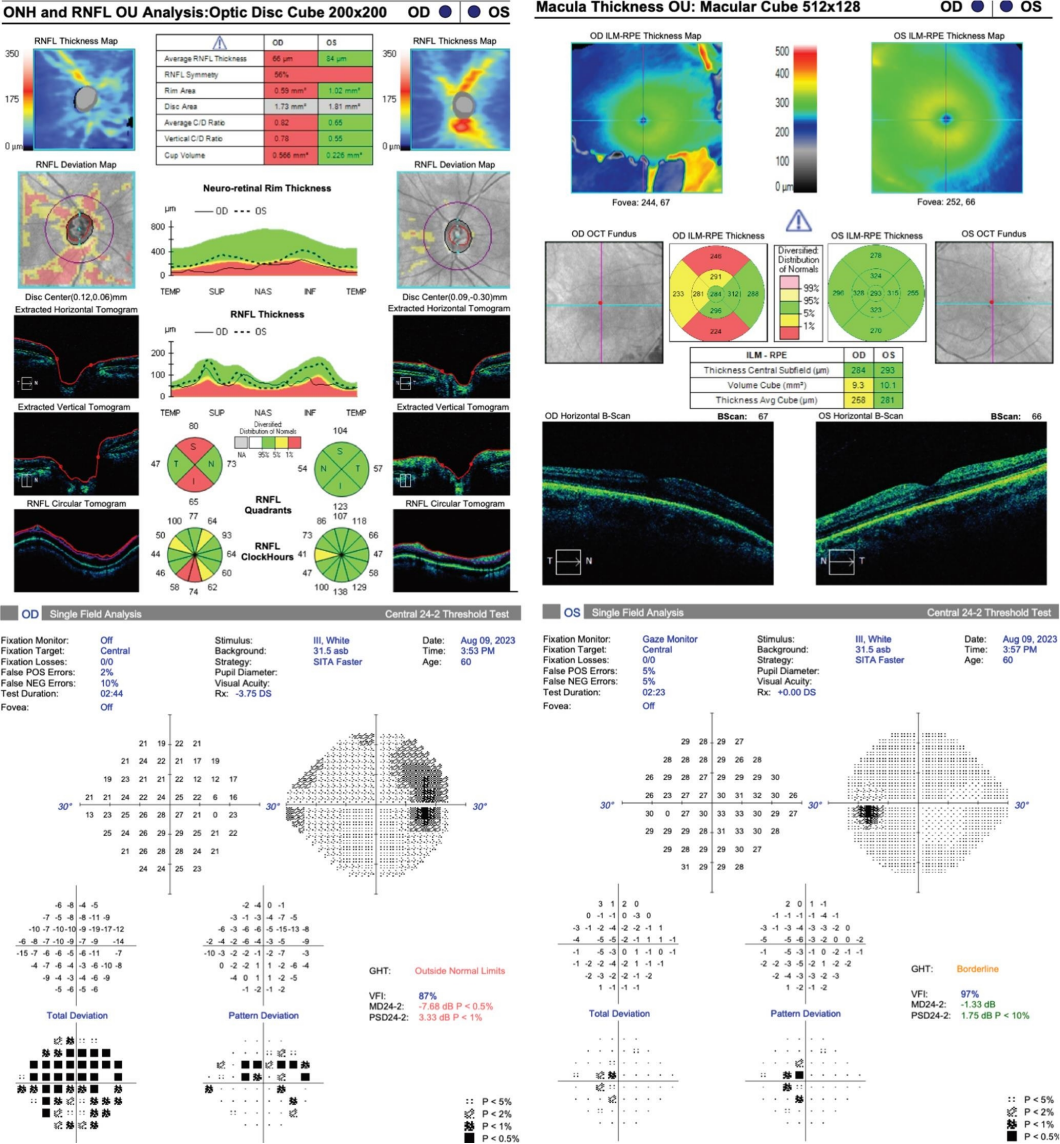
Optical Coherence tomography and Humphrey visual fields showing right glaucomatous changes.

**Figure 2:**
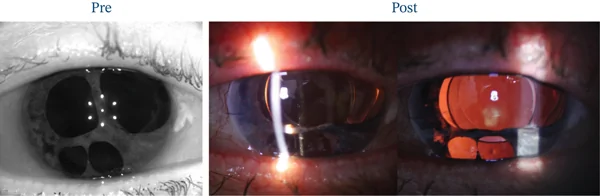
Precataract extraction photo showing essential iris atrophy with iris obscuring the central visual axis. Postcataract surgery photos demonstrate IOL placement with iris cleared from the visual axis.

**Figure 3:**
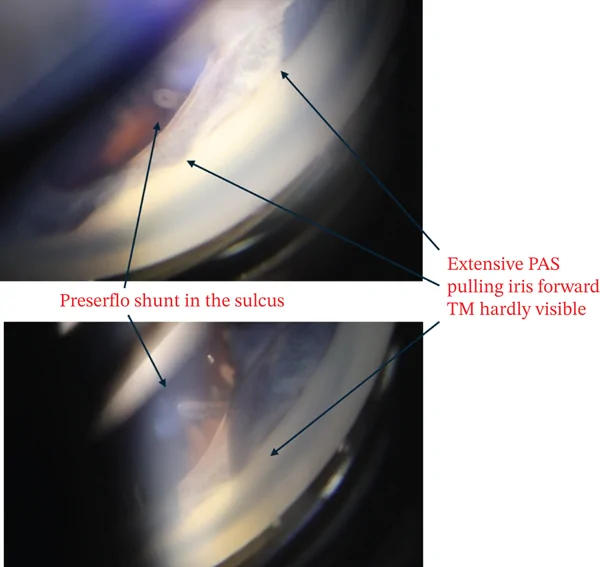
Gonioscopy showing extensive peripheral anterior synechiae with PreserFlo Microshunt in the sulcus.

Four weeks post‐PreserFlo, the IOP had improved to 6 mmHg in the right eye. Gonioscopy confirmed the presence of the PreserFlo in the sulcus. On latest review, 18 months postoperatively, her best‐corrected visual acuities were 6/12 in the right with IOPs of 16 mmHg. There was a diffuse, quiet superotemporal bleb. Her glaucoma showed no signs of progression. At this review, the once daily steroid drop was ceased given the bleb remained quiet and diffuse over successive visits.

## 3. Discussion

ICE syndrome patients frequently require surgical intervention to control their IOP. Trabeculectomy with antifibrotic agents has been commonly used; however, it is associated with high failure rates [1]. In a retrospective case series of 26 patients, trabeculectomy with antifibrotic agents had a survival rate of 29% at 5 years [1]. This high failure rate may be due to blockage of the internal ostium by abnormal endothelial membranes. In our case, the trabeculectomy failure was a result of bleb fibrosis. The bleb was shallow and fibrosed prior to the cataract surgery. Gonioscopy performed in the clinic did not show iris occlusion of the sclerotomy site and thus needling was not attempted as it was not deemed likely to be successful. Glaucoma drainage implants (GDIs) have been associated with better success rates of 53% at 5 years [1]. GDIs involve creating a bypass channel for the aqueous humour to drain from the anterior chamber to the subconjunctival space. In our case, because of extensive PAS and risk of corneal decompensation, sulcus placement was preferred. This is the first report to describe sulcus placement of the PreserFlo Microshunt in ICE syndrome.

Although GDIs have better success rates than trabeculectomy in ICE syndrome, they still frequently require repositioning of the tube because of a blocked tube ostium by iris and/or ICE membrane or anterior migration of the tube resulting in tube‐corneal touch [1, 4]. In previous studies on GDIs in ICE syndrome, 4 out of 21 patients and 5 out of 10 positions required repositioning [1, 4]. In addition, corneal decompensation postoperatively can significantly limit visual acuity. In a retrospective case series of 10 patients with ICE syndrome who underwent GDI, 7 of the 10 had corneal decompensation postoperatively resulting in limited vision [4]. Sulcus placement of the PreserFlo Microshunt may reduce the risk of tube blockage from the ICE cells, in addition to reducing the risk of corneal decompensation and tube‐corneal touch. Previous studies have shown sulcus placement of the Ahmed glaucoma valve is associated with a lower endothelial cell density loss than anterior chamber placement, and this likely extends to other GDIs, including the PreserFlo Microshunt [5].

Sulcus placement of the PreserFlo Microshunt has not been previously described in the literature. In a survey of glaucoma experts, 73% agreed that sulcus placement of the PreserFlo Microshunt may be considered in selected patients [6]. Usually, sulcus GDD is inserted bevel down to reduce risk of iris tissue blocking the entry site with physiological iris movement. In our case, the PreserFlo was inserted bevel up as there was no physiological iris movement superotemporally with iris tenting to the limbus from essential iris atrophy. We propose that ICE patients may be one group of patients where sulcus placement should be strongly considered, to reduce the risks of corneal decompensation and tube blockages. Further studies are however required to assess long‐term outcomes of patients with PreserFlo Microshunt in the sulcus.

In summary, we report the successful implantation of a PreserFlo Microshunt in the sulcus in a patient with ICE syndrome who had a previous failed trabeculectomy. The PreserFlo Microshunt in the sulcus may be preferred for ICE patients to reduce the risk of corneal decompensation and tube blockage or migration.

## Author Contributions

**Khizar Rana:** writing (original draft, review and editing). **Cindy Shi:** writing (original draft, review and editing). **Stephanie Stavrinakis:** supervision. **Soo Khai Ng:** conceptualisation, supervision and writing (review and editing).

## Funding

Open access publishing facilitated by Adelaide University, as part of the Wiley ‐ Adelaide University agreement via the Council of Australasian University Librarians.

## Disclosure

No funding, data or other resources were provided by the Vision Eye Institute for this work.

## Consent

Written consent to use this case for publication was obtained from the patient via a signed form.

## Conflicts of Interest

One author named Stephanie Stavrinakis, listed in the research group for this study, is affiliated with Vision Eye Institute, Kurralta Park. Care has been taken to report on the patient case using rationalised observation, objective evidence and soundly cited references. The named author′s affiliation with Vision Eye Institute as ‘South Australia Clinical Coordinator’ had no role in the design, conduct, analysis or interpretation of this study, nor the decision to submit the manuscript for publication.
